# Supplementary material for: Characteristic Changes and Potential Markers of Flavour in Raw Pu-Erh Tea with Different Ageing Cycles Analysed by HPLC, HS-SPME-GC-MS, and OAV
Source: Foods. 2025 Feb 27;14(5):829. doi: 10.3390/foods14050829 (PMC11898670; doi:10.3390/foods14050829)
Supplement: Supplementary file 1 [file foods-14-00829-s001.zip › foods-3464690-supplementary.pdf]

Table S1. Content of non-volatile compounds, P-value, VIP-value and Fold change of PRT with different ageing cycles

Non-volatile Compounds	Content			P-value			VIP			Fold Change		
	A1	B1	C1	A1vsB1	A1vsC1	B1vsC1	A1vsB1	A1vsC1	B1vsC1	A1vB1	A1vsC1	B1vsC1
WE(%)	42.84±2.54	44.36±2.38	45.51±0.58	0.154	0.026	0.261	0.52	0.89	0.20	1.04	1.06	1.03
TP(%)	26.65±0.15	25.7±0.51	24.87±0.24	0.046	0.000	0.054	0.75	1.17	0.89	0.96	0.93	0.97
FAA(%)	4.54±0.23	3.52±0.42	3.73±0.3	0.000	0.000	0.293	1.27	1.23	0.26	0.78	0.82	1.06
SS(%)	3.46±0.28	3.13±0.46	3.2±0.52	0.068	0.236	0.748	0.72	0.46	0.14	0.90	0.92	1.02
TR(%)	3.49±0.22	3.92±0.14	4.22±0.08	0.000	0.000	0.000	1.28	1.32	1.32	1.12	1.21	1.08
TF(%)	0.13±0.01	0.14±0.01	0.16±0.01	0.002	0.000	0.000	1.05	1.25	1.28	1.08	1.23	1.14
TB(%)	1.95±0.17	2.44±0.24	2.83±0.03	0.000	0.000	0.001	1.27	1.34	1.17	1.25	1.45	1.16
CA(%)	2.52±0.88	2.41±0.56	2.51±0.58	0.001	0.974	0.001	0.90	0.60	1.15	0.96	1.00	1.04
GCG(mg/g)	1.04±0.28	1.12±0.18	2.12±0.69	0.395	0.001	0.000	0.67	1.14	1.37	1.08	2.04	1.89
GC(mg/g)	3.54±0.42	2.61±0.33	3.17±0.48	0.000	0.142	0.006	1.22	0.49	1.21	0.74	0.90	1.21
EGCG(mg/g)	38.94±1.82	34.03±1.33	34.62±1.01	0.000	0.000	0.338	1.35	1.08	0.58	0.87	0.89	1.02
EGC(mg/g)	10.17±1.61	7.97±1.27	10.04±1.16	0.001	0.892	0.009	1.02	0.03	1.25	0.78	0.99	1.26
ECG(mg/g)	19.81±1.73	17.1±1.69	16.34±1.71	0.001	0.002	0.368	1.11	1.01	0.34	0.86	0.82	0.96
EC(mg/g)	7.84±1.3	6.88±0.21	7.37±1.61	0.023	0.546	0.318	0.93	0.63	0.55	0.88	0.94	1.07
CG(mg/g)	0.49±0.23	0.63±0.27	1.31±0.21	0.192	0.000	0.000	0.75	1.29	1.41	1.29	2.67	2.08
C(mg/g)	4.01±0.47	3.44±0.45	4.42±0.4	0.007	0.099	0.000	0.89	0.73	1.38	0.86	1.10	1.28
GA(mg/g)	1.12±0.08	1.51±0.19	1.43±0.12	0.000	0.000	0.348	1.31	1.20	0.23	1.35	1.28	0.95
Rut(mg/g)	1.33±0.21	1.51±0.15	2.59±0.64	0.020	0.000	0.000	1.01	1.20	1.36	1.14	1.95	1.72
Que(mg/g)	0.33±0.06	0.56±0.37	1.7±0.58	0.091	0.000	0.000	0.66	1.28	1.49	1.70	5.15	3.04
Myr(mg/g)	0.06±0.02	0.06±0.02	0.04±0.01	0.483	0.013	0.113	0.78	0.76	0.61	1.00	0.67	0.67
Kea(mg/g)	0.01±0.00	0.01±0.00	0.02±0.01	0.339	0.477	0.128	0.83	0.37	0.51	1.00	2.00	2.00
Theo(mg/g)	0.14±0.01	0.1±0.01	0.07±0.01	0.000	0.006	0.029	1.08	1.34	0.83	0.71	0.50	0.70

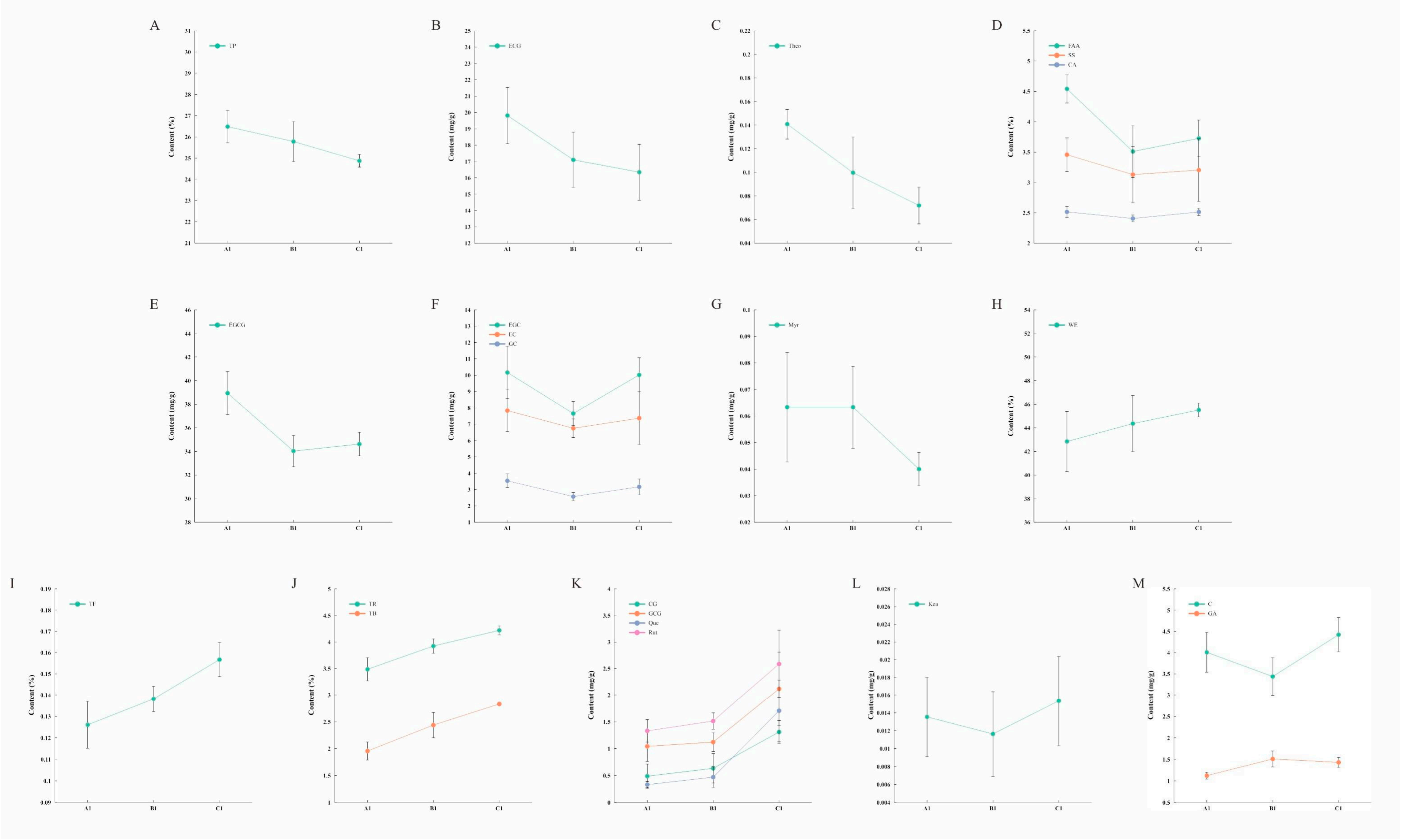

Figure S1. Changes in the content of non-volatile compounds in RPT with different ageing cycles. (A) Tea polyphenols. (B) (−)-epicatechin gallate. (C) Theophylline. (D) Free amino acids, soluble sugars and caffeine. (E) (−)-epigallocatechin gallate. (F) (−)-epigallocatechin, (−)-epicatechin, (+)-gallocatechin. (G) Myrcene. (H) Water extracts. (I) Theaflavins. (J) Thearubigins, and theabrownins. (K) (−)-catechin gallate, (−)-gallocatechin gallate, quercetin and rutin. (L) Kaempferol. (M) (+)-catechin and gallic acid.

Table S2. Sum of the absolute contents of volatile compounds in PRT with different ageing years

Types of volatile compounds	Absolute Content(μg/L)									
	Y1	Y2	Y3	Y4	Y5	Y6	Y7	Y8	Y9	Y10
Alkenes	316.81	902.11	1533.84	470.00	285.16	347.65	514.84	855.74	357.24	233.12
Alcohols	215.86	591.05	1410.99	571.98	211.10	330.10	216.75	707.86	462.22	369.40
Esters	243.68	499.30	1628.21	366.52	197.55	291.13	190.31	691.99	307.75	389.91
Alkanes	274.41	914.07	1148.93	416.66	212.79	186.05	177.80	476.69	171.73	185.80
Aldehydes	441.49	583.65	1606.15	152.32	104.56	46.51	145.06	554.97	259.61	141.61
Ketones	305.43	563.55	926.72	233.73	195.96	200.34	216.81	413.63	164.46	220.19
Aromatic hydrocarbons	292.91	604.32	701.60	113.57	168.20	104.46	220.20	261.17	102.32	132.07
Heterocycles	166.10	214.19	620.14	16.31	55.37	12.60	30.62	107.96	66.73	55.26
Others	12.10	82.57	423.47	155.52	0.00	6.17	0.00	20.11	0.00	9.48

Table S3. OAV of key aroma-active compounds of RPT with different ageing years

Aroma Compounds	RI	CAS	Thresholds (μg/L)	OAV(μg/L)										Aroma Characteristics	Class
				Y1	Y2	Y3	Y4	Y5	Y6	Y7	Y8	Y9	Y10		
Linalool	1100.49	78-70-6	0.006	15289.53	3875.95	106598.48	61178.99	10938.13	18538.13	13653.52	46010.78	27321.07	13991.96	Floral	Floral
Safranal	1199.86	116-26-7	0.003	ND	ND	73552.80	15509.35	ND	ND	ND	38114.63	16283.67	ND	Saffron, herbal, woody	Herbal
β -Ionone	1490.58	14901-07-6	0.0059	27602.19	54986.41	74251.90	19473.98	12348.77	18699.06	13557.37	32479.56	13331.94	14141.78	Violet, floral, sweet, fruity, woody	Floral
β -Cyclocitral	1221.65	432-25-7	0.003	26525.52	42825.72	87428.53	14667.45	ND	13286.80	ND	28624.11	13260.78	11234.41	Sweet, fruity, herbaceous	Sweet
Copaene	1416.13	3856-25-5	0.006	12872.69	18794.08	28492.76	11848.38	ND	11648.83	ND	16643.16	ND	ND	Honey, spicy, woody	Sweet
D-Limonene	1027.00	5989-27-5	10	2078.29	4916.65	14880.11	3797.51	4188.66	2586.49	5667.77	9114.03	4470.05	2902.84	Fruity, sweet, lemon, orange	Fruity
p-Cymene	1023.31	99-87-6	0.0114	ND	ND	8936.24	ND	ND	ND	4294.56	7121.33	ND	ND	Citrus,petrol	Fruity
Heptanal	901.81	111-71-7	0.26	ND	ND	9890.26	1901.52	ND	ND	1343.59	6225.82	ND	ND	Almond, nuts	Nuts
Hexanal	819.83	66-25-1	5	2025.66	ND	ND	2420.37	ND	ND	1921.62	4072.02	1904.64	1479.57	Herbaceous, fruity	Herbal
Limonene	1030.09	138-86-3	0.01	ND	ND	ND	ND	ND	ND	ND	3383.37	ND	ND	Fruity, berries	Fruity
Nonanal	1104.35	124-19-6	1	3067.79	3943.61	10023.89	ND	1045.56	ND	1209.17	3101.28	1341.95	1005.07	Light and sweet rose aroma, citrus	Floral
1-Methyl-naphthalene	1324.70	90-12-0	0.008	ND	ND	ND	ND	0.00	ND	3261.42	2937.43	745.36	ND	Camphor, herbal aroma, chemical	Herbal
(+)- α -Pinene	931.35	7785-70-8	0.0053	ND	ND	0.00	ND	0.00	ND	ND	2462.64	ND	ND	Strong wood and pine	Woody

1-Octen-3-ol	983.79	3391-86-4	1	ND	ND	16220.49	785.48	0.00	ND	ND	2328.70	ND	ND	Greasy, mushroom, floral	Green
Trans- β -Ocimene	1038.05	3779-61-1	0.034	ND	ND	1261.72	ND	308.64	ND	379.37	710.27	295.95	ND	Sweet, herbal	Sweet
(Z)-3,7-Dimethylocta-1,3,6-triene	1048.50	3338-55-4	0.034	157.27	917.32	2024.92	197.79	230.74	129.29	458.18	694.31	581.93	135.47	Floral, herbal, sweet	Floral
2,2,6-Trimethyl-cyclohexanone	1033.39	2408-37-9	0.1	418.24	425.01	1834.61	371.83	224.34	120.21	281.25	636.57	342.32	192.06	Honey, black pepper	Sweet
Dibutyl phthalate	1973.02	84-74-2	0.26	90.57	186.85	1255.75	139.87	ND	ND	ND	598.55	ND	177.35	Coffee	Roasted
(E)-Linalool oxide (furanoid)	1073.89	34995-77-2	0.06	ND	ND	ND	211.87	ND	ND	ND	541.16	378.06	ND	Woody, floral	Woody
(Z)-linalool oxide (furanoid)	1074.08	5989-33-3	0.32	ND	ND	ND	ND	66.90	71.96	95.43	538.16	160.01	271.42	Strong woody and floral, camphor	Woody
(±)-Dihydroactinidiolide	1547.93	15356-74-8	0.5	18.20	79.79	971.25	264.82	182.73	208.46	124.38	466.04	193.65	279.60	Fruity, ripe apricot, oily, woody	Fruity
β -Caryophyllene	1422.65	87-44-5	1.54	520.58	2541.44	751.12	ND	ND	ND	ND	419.70	ND	ND	Warm woody notes, violet, orris root	Woody
Cedrol	1610.72	77-53-2	0.5	125.46	387.34	704.85	148.77	241.78	269.63	130.79	356.77	318.29	394.76	Woody	Woody
α -Terpineol	1194.71	98-55-5	0.3	ND	120.09	ND	152.08	44.69	63.07	93.73	319.06	235.17	56.95	Orchid, light, fruity, banana	Green
α -Terpinene	1015.17	99-86-5	7.9	ND	ND	537.72	111.18	ND	ND	151.40	279.74	108.36	108.80	Woody, citrus	Woody
Terpinolene	1087.07	586-62-9	0.2	45.26	164.63	ND	96.92	101.04	131.83	213.61	244.86	114.09	61.97	Herbal, woody	Herbal
Naphthalene	1185.88	91-20-3	0.5	253.71	239.50	481.36	99.83	144.49	59.70	99.09	132.80	88.20	66.60	Camphor, tar	Herbal
γ -Terpinene	1058.38	99-85-4	2.1	8.57	38.41	174.85	19.72	26.97	12.65	30.06	60.88	27.87	13.06	Citrus, lemon	Floral
Terpinen-4-ol	1179.44	562-74-3	1.2	ND	ND	ND	14.04	ND	8.93	7.30	24.89	14.37	11.14	Woody	Woody
Benzaldehyde	967.02	100-52-7	0.085	ND	ND	64.63	ND	ND	ND	5.67	15.95	29.16	ND	Nuts, bitter almond	Nuts
Farnesol	1590.27	4602-84-0	1	13.63	ND	48.80	9.92	11.18	10.01	3.67	14.39	ND	13.33	Sweet, floral, green	Sweet
Dihydrocarvone	1169.53	7764-50-3	3.25	ND	ND	ND	ND	ND	ND	ND	3.96	ND	ND	Herbal, like spearmint	Herbal
Eucalyptol	1029.90	470-82-6	2	6153.00	8519.21	23073.88	5877.94	ND	4144.04	6164.57	ND	5264.38	5430.59	Mint	Green
2-Amylfuran	990.61	3777-69-3	0.006	4201.53	10085.58	15656.58	ND	ND	ND	ND	ND	ND	ND	roasted, sweet fruity	Roasted
2-Methyl-naphthalene	1305.15	91-57-6	0.004	1809.95	10963.48	ND	ND	ND	1799.20	9116.92	ND	ND	ND	Sweet, floral, woody	Sweet
2-Methyl-butanal	779.84	96-17-3	0.001	7412.61	ND	ND	4380.33	ND	ND	ND	ND	4502.00	ND	Nuts, grains	Nuts
cis-Anethol	1302.87	104-46-1	0.015	ND	421.88	3719.92	2315.39	ND	ND	ND	ND	ND	ND	Herbal, aniseed, sweet	Herbal
Phenylacetaldehyde	1051.79	122-78-1	0.0063	ND	ND	4541.08	ND	ND	ND	ND	ND	668.10	ND	Hyacinth,sweet	Floral

Estragole	1313.78	140-67-0	0.035	ND	ND	4146.70	ND	ND	ND	ND	ND	ND	ND	Herbal	Herbal
2-Carene	1087.45	554-61-0	0.77	ND	ND	129.70	ND	14.58	ND	ND	ND	ND	ND	Wood, pine	Woody
3-Carene	1102.20	13466-78-9	9.3	ND	107.62	ND	ND	ND	ND	ND	ND	ND	ND	Pine	Woody
Methyl palmitate	1929.35	112-39-0	2	ND	ND	21.23	ND	ND	ND	ND	ND	ND	0.63	Wax, iris	Fatty
o-Cymene	1023.12	527-84-4	11.4	0.70	1.74	ND	2.48	2.21	1.63	ND	ND	3.61	2.27	Floral	Floral
3-Nonen-2-one	1150.39	14309-57-0	0.03	11.61	ND	ND	ND	ND	ND	ND	ND	ND	ND	Fruity, pungent, liquorice	Fruity

Table S4.OAV, P-value, VIP-value and Fold Change of key aroma-active compounds with different ageing cycles

Aroma Compounds	OAV(μg/L)			P-value			VIP			Fold Change		
	A2	B2	C2	A2	B2	C2	A2vsB2	A2vsC2	B2vsC2	A2vsB2	A2vsC2	B2vsC2
α-Terpineol	40.03	88.39	203.73	0.317	0.133	0.162	0.63	1.55	2.07	2.21	5.09	2.3
1-Methyl-naphthalene	ND	815.36	1227.6	0.437	0.128	0.148	0.42	0.63	0.24	15.45	23.26	1.51
Heptanal	3296.75	811.28	3001.4	0.418	0.941	0.232	0.41	0.05	0.84	0.25	0.91	3.7
Farnesol	20.81	8.7	9.24	0.371	0.49	0.905	0.57	0.54	0.14	0.42	0.44	1.06
Terpinen-4-ol	ND	7.57	16.8	0.079	0.016	0.117	0.69	1.14	1.25	21.51	47.75	2.22
β-Cyclocitral	52259.92	6988.56	17706.43	0.036	0.143	0.167	2.62	2.23	2.1	0.13	0.34	2.53
α-Terpinene	179.24	65.65	165.63	0.501	0.946	0.191	0.4	0.05	0.94	0.37	0.92	2.52
Copaene	20053.18	5874.3	5547.72	0.05	0.113	0.96	2.1	2.1	0.02	0.29	0.28	0.94
(Z)-linalool oxide (furanoid)	ND	58.57	323.2	0.06	0.045	0.041	0.59	1.37	2.12	13.87	76.55	5.52
cis-Anethol	1380.6	578.85	ND	0.533	0.306	0.437	0.85	1.68	1.58	0.42	0.01	0.01
Nonanal	5678.43	563.68	1816.1	0.041	0.166	0.119	5.07	4.22	4.08	0.1	0.32	3.22
(E)-Linalool oxide (furanoid)	ND	52.97	306.41	0.437	0.236	0.748	0.25	0.96	1.41	7.53	43.54	5.78
D-Limonene	7291.68	4060.11	5495.64	0.377	0.698	0.445	1.72	0.94	1.8	0.56	0.75	1.35
Eucalyptol	12582.03	4046.64	3564.99	0.131	0.182	0.839	1.06	1.1	0.21	0.32	0.28	0.88
Safranal	24517.6	3877.34	18132.77	0.371	0.824	0.226	1.23	0.4	2.21	0.16	0.74	4.68
Dibutyl phthalate	511.061	34.97	258.64	0.191	0.574	0.207	2.09	1.17	2.66	0.07	0.51	7.4
2-Carene	43.23	3.65	ND	0.327	0.374	0.437	0.95	1.03	0.45	0.08	0.01	0.15
2-Methyl-naphthalene	4257.81	2729.03	ND	0.706	0.278	0.337	0.15	0.7	0.96	0.64	0.03	0.05
Linalool	41921.32	26077.19	29107.94	0.626	0.724	0.857	1.09	1.01	0.61	0.62	0.69	1.12
(Z)-3,7-Dimethylocta-1,3,6-triene	1033.17	254	470.57	0.152	0.378	0.248	1.03	0.78	0.84	0.25	0.46	1.85
2,2,6-Trimethyl-cyclohexanone	892.62	249.41	390.32	0.168	0.362	0.313	1.5	1.28	1.13	0.28	0.44	1.56
γ-Terpinene	73.94	22.35	33.94	0.285	0.493	0.403	1.27	1	0.94	0.3	0.46	1.52
Cedrol	405.88	197.74	356.61	0.213	0.785	0.016	1.9	0.51	0.51	0.49	0.88	1.8
1-Octen-3-ol	5406.83	196.37	776.23	0.302	0.444	0.439	0.99	0.95	0.57	0.04	0.14	3.95
(±)-Dihydroactinidiolide	356.41	195.1	313.1	0.562	0.898	0.179	1.24	0.33	2.81	0.55	0.88	1.6

Trans-β-Ocimene	420.57	172	335.41	0.533	0.865	0.47	0.42	0.14	0.59	0.41	0.8	1.95
β-Ionone	52280.17	16019.8	19984.43	0.025	0.096	0.514	3.32	3.16	1.3	0.31	0.38	1.25
Terpinolene	69.96	135.85	140.31	0.262	0.391	0.939	0.73	0.66	0.03	1.94	2.01	1.03
2-Methyl-butanal	2470.87	1095.08	1500.67	0.597	0.754	0.831	0.32	0.1	0.08	0.44	0.61	1.37
Hexanal	675.22	1085.5	2485.41	0.681	0.159	0.223	0.15	0.65	0.86	1.61	3.68	2.29
p-Cymene	2978.75	1073.64	2373.78	0.526	0.881	0.605	0.61	0.2	0.79	0.36	0.8	2.21
Naphthalene	324.86	100.785	95.87	0.022	0.047	0.859	2.48	2.58	0.24	0.31	0.3	0.95
o-Cymene	0.81	1.58	1.96	0.372	0.382	0.744	0.51	0.65	0.42	1.95	2.42	1.24
Benzaldehyde	21.54	1.42	15.04	0.317	0.792	0.119	0.45	0.15	0.79	0.07	0.7	10.61
β-Caryophyllene	1271.051	ND	139.9	0.063	0.159	0.286	1.92	1.84	0.97	0.01	0.11	21.21
Phenylacetaldehyde	1513.69	ND	222.7	0.286	0.387	0.286	0.55	0.47	0.37	0.04	0.15	3.32
Methyl palmitate	7.08	ND	0.21	0.286	0.374	ND	0.64	0.65	0.21	0.03	0.03	1
Limonene	ND	ND	1127.79	ND	0.374	0.286	ND	0.6	1.09	ND	26.71	26.71
